# Antiangiogenesis and gene aberration-related therapy may improve overall survival in patients with concurrent *KRAS* and *TP53* hotspot mutant cancer

**DOI:** 10.18632/oncotarget.16840

**Published:** 2017-04-05

**Authors:** Zhijie Wang, Sarina Piha-Paul, Filip Janku, Vivek Subbiah, Naiyi Shi, Jing Gong, Chetna Wathoo, Kenna Shaw, Kenneth Hess, Russell Broaddus, Aung Naing, David Hong, Apostolia M. Tsimberidou, Daniel Karp, James Yao, Funda Meric-Bernstam, Siqing Fu

**Affiliations:** ^1^ Department of Investigational Cancer Therapeutics, The University of Texas MD Anderson Cancer Center, Houston, Texas, USA; ^2^ Institute of Personalized Cancer Therapy, The University of Texas MD Anderson Cancer Center, Houston, Texas, USA; ^3^ Department of Biostatistics, The University of Texas MD Anderson Cancer Center, Houston, Texas, USA; ^4^ Department of Pathology, The University of Texas MD Anderson Cancer Center, Houston, Texas, USA; ^5^ Department of Gastrointestinal Medical Oncology, The University of Texas MD Anderson Cancer Center, Houston, Texas, USA; ^6^ National Cancer Center/Cancer Hospital, Chinese Academy of Medical Science and Peking Union Medical College, Beijing, China

**Keywords:** KRAS, TP53, chronic inflammation, phase I trial, gene aberration-related therapy

## Abstract

**Purpose:**

Genetic alterations such as activating *KRAS* and/or inactivating *TP53* are thought to be the most common drivers to tumorigenesis. Therefore, we assessed phase I cancer patients with *KRAS+*/*TP53+* mutations.

**Results:**

Approximately 8% of patients referred to phase I clinical trials harbored concurrent *KRAS* and *TP53* mutations. Patients who received a phase I trial therapy (n = 57) had a median OS of 12 months, compared with 4.6 months in those who were not treated (n = 106; *p* = 0.003). *KRAS* G13 and *TP53* R273 mutations were associated with poor overall survival (OS), while antiangiogenesis and gene aberration-related therapies were associated with prolonged OS. A prognostic model using neutrophilia, thrombocytosis, hypoalbuminemia, body mass index <30 kg/m^2^, and the absence of lung metastasis was established and validated. Phase I cancer patients in the low-risk group had a median OS of 16.6 months compared with 5.4 months in the high-risk group (*p* < 0.001). Untreated patients in the low-risk group had a median OS of 6.7 months compared with 3.6 months in the high-risk group (*p* = 0.033).

**Experimental Design:**

We analyzed 163 consecutive patients with advanced *KRAS+*/*TP53+* mutant cancer who were referred to phase I clinical trials, to identify molecular aberrations, clinical characteristics, survivals, and potentially effective treatment regimens.

**Conclusions:**

This study provided preliminary evidence that besides modulation of the proinflammatory state, antiangiogensis and concomitant gene aberration-related therapies may improve the treatment of *KRAS+*/*TP53+* mutant cancer.

## INTRODUCTION

Oncogenic mutations in rat sarcoma viral oncogene homolog (*RAS*) genes are detected in approximately 30% of human cancers, predominantly in colorectal cancer, pancreatic cancer, and lung adenocarcinomas [1]. These mutations occur most frequently in Kirsten *RAS* (*KRAS*), which encodes a small GTPase that mediates downstream signaling from growth factor receptors [2, 3]. *KRAS* mutations can constructively activate downstream signaling pathways, such as *RAS*/mitogen-activated protein kinase (MEK)/extracellular signal-regulated kinases (ERK) and phosphoinositide 3-kinase (PI3K)/AKT/mammalian target of rapamycin (mTOR), and this signaling pathway activation triggers nuclear gene transcription and cell differentiation and proliferation [4].

However, *KRAS* mutation alone, which occurs in the early process of tumorigenesis, is not sufficient to induce malignant transformation of normal epithelial cells [5, 6]. Additional loss of tumor suppressor genes, such as *TP53* [7, 8], is required for cancer development, which arises through sequential accumulation of oncogenic mutations and loss of tumor suppressor genes. Somatic *TP53* mutation is the most common genetic aberration in tumor suppressor genes, occurring in 10% to 96% of human cancers [9]. Functional *TP53* mutations lead to ablation of cell cycle arrest and DNA damage repair, as well as overexpression of nuclear target genes, resulting in genomic instability and tumor development [10]. Dual mutations in *TP53* and *KRAS* (*KRAS+*/*TP53+*, + indicates positive hotspot test) occur in up to 20% of advanced solid tumors [11–14]. In genetically engineered mouse models, mice harboring both the *TP53* R172H and *KRAS* G12D mutations had a significantly shortened latency, and thus more tumors than mice with the *KRAS* G12D mutation alone [7, 15].

Because concurrent *KRAS* and *TP53* mutations manifest potentially synergistic biologic effects, cancers carrying both *KRAS* and *TP53* mutations (*KRAS*+/*TP53*+) might represent a unique cancer subtype with distinct and aggressive biologic behaviors [16]. Blockade of downstream signaling pathways such as RAF/MEK or PI3K/AKT/mTOR in *KRAS*-mutant cancer [17] and antiangiogenic-based therapy in *TP53*-mutant cancer would be appropriate therapeutic strategies [18, 19]. Unfortunately, effective therapies directly targeting *TP53* or *KRAS* mutations are not available and these mutations are currently considered undruggable [20, 21].

Many phase I clinical trials include patients with malignancies arising from undruggable genetic mutations, but it is unclear which types of therapies are most promising for the treatment of these malignancies. Therefore, it is of great scientific interest and clinical urgency to explore potential therapeutic options for malignancies with undruggable genetic mutations. In the current study, we reviewed demographics and clinical outcomes of patients with advanced *KRAS+*/*TP53+* mutant cancers who were referred to phase I clinical trials at The University of Texas MD Anderson Cancer Center. Our aims were to investigate specific genetic aberrations associated with clinical outcomes and to identify potential therapeutic regimens for the treatment of advanced *KRAS+*/*TP53+* mutant cancers.

## RESULTS

### Patient characteristics

From March 2102 to October 2014, 2, 144 consecutive patients with advanced cancers were referred to phase I clinical trials at MD Anderson and underwent molecular tests for tumor genetic aberrations. Among these patients, 167 (7.8%) harbored concurrent *KRAS* and *TP53* hotspot mutations (*KRAS+*/*TP53+* mutant cancer), 182 (8.5%) harbored *KRAS+*/*TP53*– hotspot mutations, and 839 (39.1%) harbored *KRAS*–/*TP53+* hotspot mutations (- indicates negative hotspot test). Four patients with *KRAS+*/*TP53+* mutant cancer had insufficient clinical data and were not included in our analysis. The baseline characteristics of the remaining 163 patients are summarized in Table 1.

**Table 1:** Patient baseline characteristics (n=163)

Characteristics	Patient number	Percentage (%)
Age (median, range)	55 (17-83)	
Gender		
Male	97	60
Female	66	40
Race		
White	103	63
African American	25	15
Hispanic	23	14
Asian	4	3
Others	8	5
Type of cancer		
Colorectal	104	64
Pancreatic	28	17
Lung*	8	5
Others**	23	14
With second primary cancer		
Yes	20	12
No	143	88
Sites of metastasis		
Lung	116	71
Liver	113	69
Lymph node	61	37
Peritoneal	37	23
Bone	23	14
Retroperitoneal	20	12
Adrenal	17	10
Soft tissue	12	7
Brain	8	5
Cutaneous	7	4
Renal	6	4
Spleen	6	4
Ovarian	4	2
Vaginal	4	2
Initial diagnosis with metastasis		
Yes	89	55
No	74	45

^*^Lung cancers included adenocarcinoma (n=5), adenosquamous (n=2) and neuroendocrine (n=1). **Other cancers included cholangiocarcinoma (n=3), esophageal (n=1), gastric (n=1), duodenal (n=1), uterine (n=4), ovarian (2), vaginal (n=1), bladder (n=1), sinonasal (n=1), thyroid (n=1), appendiceal (n=2), skin squamous (n=1) and cancer of unknown primary (n=4).

### Molecular aberrations

In the 163 patients with *KRAS+*/*TP53+* mutant cancer, G12 (n = 107; 66%) and G13 (n = 25; 15%) mutations constituted the majority of *KRAS* hotspot mutations. In patients with pancreatic cancers, G12 mutations occurred more frequently (*p* = 0.003), but G13 mutations were not found. In the total cohort of patients (n = 163), 83 types of *TP53* mutations were found, of which 44% were common hotspot mutations: R273 (n = 26; 16%), R175 (n = 19; 12%), R248 (n = 12; 7%), G245 (n = 9; 6%), and R282 (n = 5; 3%). Association of a *TP53* hotspot mutation with a specific cancer was not observed. Other concurrent genetic aberrations were found in most patients (n = 125; 77%), and more than one concomitant genetic aberration was found in 87 patients (53%): *APC* (n = 65; 40%), *PIK3CA* (n = 37; 23%), *KIT* (n = 34; 21%), *SMAD4* (n = 18; 11%), *FBXW7* (n = 11; 7%), *MET* (n = 10; 6%), *JAK3* (n = 9; 6%), *CDKN2A* (n = 9; 6%), *PTEN* (n = 6; 4%), and *STK11* (n = 5; 3%).

### Antitumor activity and PFS

Approximately one-third of patients (n = 57) received a total of 78 phase I trial therapies under 50 different phase I clinical trials. These therapies yielded 2 PRs and 17 SDs (24% of disease control), associated with a median PFS of 2.1 months (95% confidence interval [CI] 1.8-2.4). Among patients treated with an antiangiogenic agent (n = 15), 11 (73%) had PR or SD and the median PFS was 3.7 months (95% CI 2.8-4.6), which was significantly better than among patients who were not treated with an antiangiogenic agent (8/39 [21%] PR or SD, *p* < 0.001; PFS 1.8 months [95% CI 1.6-2.0], *p* = 0.043). In patients who received therapies with one agent targeting a concomitant genetic aberration or its downstream proteins (gene aberration-related therapy), the disease control rate was 65% (17/26) and the median PFS was 3.7 months (95% CI 2.6-4.8), which was significantly better than among patients who did not receive this type of treatment (2/28 [7%] PR or SD, *p* < 0.001; PFS 1.6 months [95% CI 1.2-2.0], *p* < 0.001). In patients receiving gene aberration-related phase I clinical trial therapy, PFS was similar to that observed with previous standard of care therapy before phase I clinical trial referral (2.5 months [95% CI 1.4-3.6], *p* = 0.866).

### Overall survival

A median OS of 6.7 months (95% CI 4.9-8.5) was observed in the 163 patients with *KRAS+*/*TP53+* mutant cancer who were referred to phase I clinical trials at MD Anderson. Patients who received therapy in a phase I clinical trial had a median OS of 12 months (95% CI 5.6-18.4), which was significantly better than the median OS in those who did not (4.6 months [95% CI 3.6-5.6], *p* = 0.003). Patients receiving phase I clinical trial therapies with an antiangiogenic agent had a median OS of 13.4 months (95% CI 5.5-20.2), and those receiving gene aberration-related phase I clinical trial therapies had a median OS of 13.5 months (95% CI 5.3-20.6). These OS times compared favorably with those of patients who did not receive these treatments (no antiangiogenic therapy: median OS 8.8 months [95% CI 3.0-14.6], *p* = 0.6; and no gene aberration-related phase I clinical trial therapy: median OS 7.6 months [95% CI 7.1-8.1], *p* = 0.2) respectively.

### Association of OS with genetic aberrations

**Figure 1:**
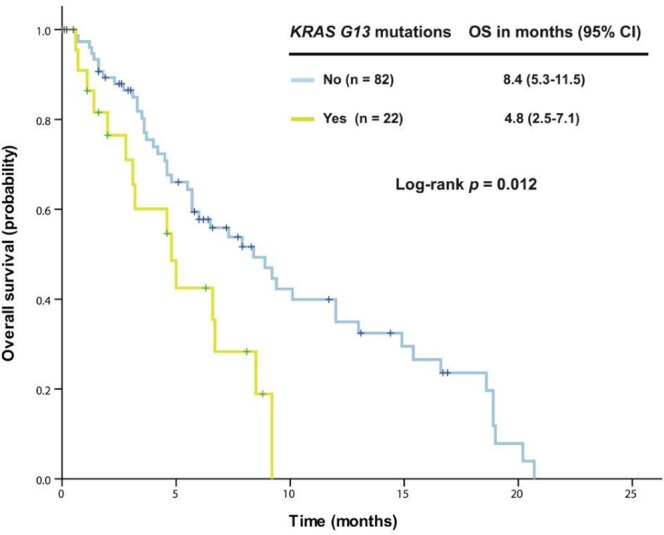
Kaplan-Meier overall survival (OS) curves in patients with *KRAS+*/*TP53+* mutant colorectal cancer who received therapy in a phase I clinical trial, stratified by *KRAS* G13 mutation status (due to sample size, all p values are unadjusted) .

**Figure 2:**
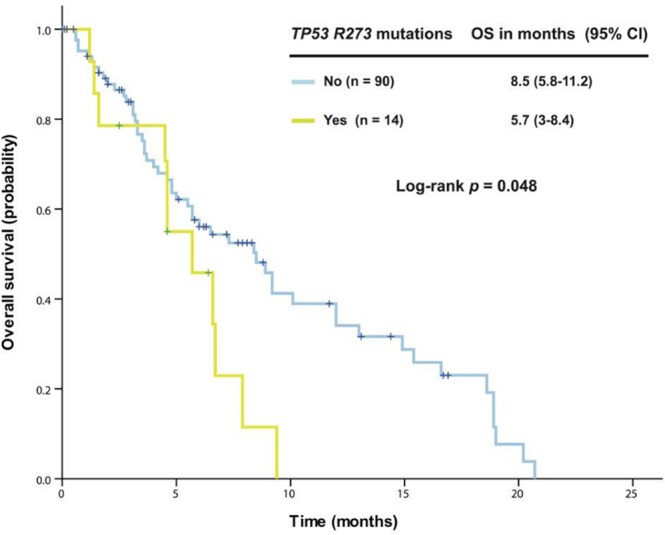
Kaplan-Meier overall survival (OS) curves in patients with *KRAS+*/*TP53+* mutant colorectal cancer who received therapy in a phase I clinical trial, stratified by *TP53* R273 mutation status (due to sample size, all p values are unadjusted) .

Further analysis in 163 patients with *KRAS+*/*TP53+* mutant cancer revealed that patients harboring G13 mutations (n = 25) had a median OS of 4.8 months (95% CI 2.5-7.1), which was significantly worse than among those without the G13 mutation (n = 138, median OS 7.3 months [95% CI 4.8-9.8], *p* = 0.016). No survival difference was observed between patients with G12 mutations and those without. In patients with colorectal cancers, G13 mutations remained associated with reduced OS (n = 22, median OS 4.8 months [95% CI 2.5-7.1]) compared with patients without G13 mutations (n = 82, median OS 8.4 months [95% CI 5.3-11.5]; *p* = 0.012), as shown in Figure 1. Patients with a *TP53* R273 mutation (n = 14) had a median OS of 5.7 months [95% CI 3.0-8.4], which was worse than in patients without the R273 mutation (n = 90, median OS 8.5 months [95% CI 5.8-11.2]; *p* = 0.048), as shown in Figure 2.

### Exploratory study of a prognostic model

**Figure 3:**
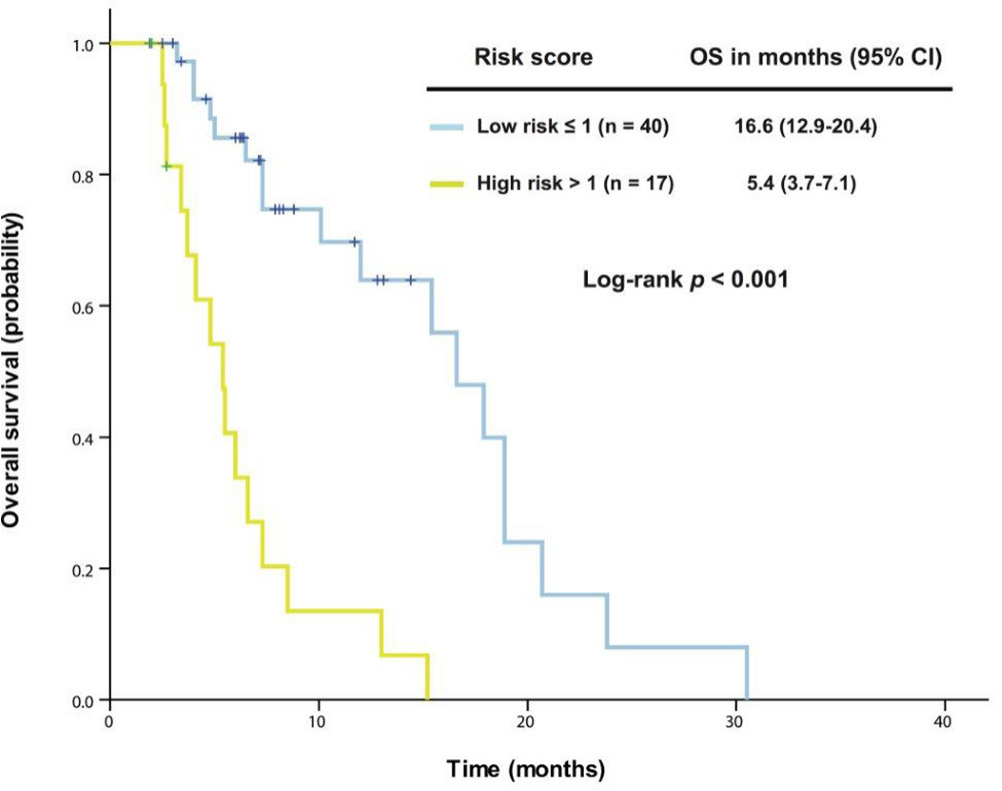
A prognostic model was established from 57 patients with advanced *KRAS+*/*TP53+* mutant cancer who received therapy in a phase I clinical trial Kaplan-Meier overall survival (OS) curves are shown, stratified by risk score (low-risk group: score ≤1, high-risk group: score >1) (due to sample size, all p values are unadjusted).

We were unable to apply the Royal Marsden Hospital score [22] or the MD Anderson prognostic score [23] to the 57 patients who received therapy in a phase I clinical trial. Therefore, we decided to explore a prognostic model specific to patients with *KRAS+*/*TP53+* mutant cancer. First, we analyzed the association of OS with potential risk factors using univariate and multivariate analyses in these 57 patients (Table 2). Five independent poor risk factors were identified for predicting individual survival outcome: neutrophilia, thrombocytosis, hypoalbuminemia, body mass index <30 kg/m^2^, and the absence of lung metastasis. These parameters were then extracted using binary subgroups (no = 0, yes = 1) to explore a risk prognostic model predictive of OS after the initial phase I clinical trial visit. This model classified the patients into one of two risk cohorts (*p* < 0.001; Figure 3): a low-risk group (score ≤ 1, n = 40) associated with a median OS of 16.6 months (95% CI 12.9-20.4) or a high-risk group (score > 1, n = 17) associated with a median OS of 5.4 months (95% CI 3.7-7.1).

**Table 2:** Univariate and multivariate analyses of OS in 57 patients who received a phase I trial therapy

Potential Risk Factors	Patient Number	Median OS (months, 95% CI)	*p* value	
			Univariate	Multivariate
Age < 65 years	Yes (n=46)	10.1 (5.2-15)	0.121	0.185
	No (n=11)	17.9 (6.7-29.1)		
Male	Yes (n=37)	15.2 (6.3-24.1)	0.334	0.228
	No (n=20)	12 (4.1-19.9)		
Colorectal cancer	Yes (n=39)	12 (6.6-17.4)	0.773	0.582
	No (n=18)	7.3 (0-16.5		
Presence of a second primary cancer	Yes (n=5)	10.1 (0, infinity)	0.086	0.106
	No (n=52)	13 (4.6-21.4)		
Metastasis at initial diagnosis	Yes (n=28)	7.3 (4.2-10.4)	0.176	0.978
	No (n=29)	13 (8-18.1)		
Number of metastatic sites≤ 2	Yes (n=18)	13 (2.1-23.9)	0.572	0.402
	No (n=39)	10.1 (4.2-16)		
Lung metastasis	Yes (n=43)	15.4 (8.3-22.5)	**0.015**	**0.01**
	No (n=14)	7.3 (4.5-10.1)		
Liver metastasis	Yes (n=41)	8.5 (4.6-12.4)	0.571	0.593
	No (n=16)	15.2 (7-23.4)		
Eastern Cooperative Oncology Group (ECOG) performance status of 0	Yes (n=8)	16.6 (3.1-30.1)	0.077	0.66
	No (n=49)	10.1 (3.1-17.1)		
Neutrophilia	Yes (n=6)	3.4 (2-4.8)	**<0.001**	**<0.001**
	No (n=51)	13 (6.6-19.4)		
Lymphopenia	Yes (n=16)	7.3 (1.9-12.7)	0.103	0.386
	No (n=41)	13 (7.4-18.6)		
Anemia	Yes (n=41)	13 (4.8-21.2)	0.778	0.097
	No (n=16)	10.1 (3.9-16.3)		
Thrombocytosis	Yes (n=1)	2.6 (0, infinity)	**<0.001**	**0.022**
	No (n=56)	12 (5.5-18.5)		
Normal lactate dehydrogenase	Yes (n=33)	15.2 (11.3-19.1)	0.11	0.119
	No (n=24)	6.5 (5-8)		
Hypoalbuminemia	Yes (n=2)	2.5 (0, infinity)	**0.016**	**0.029**
	No (n=55)	13 (6.2-19.8)		
Normal creatinine	Yes (n=56)	12 (5.6-18.4)	0.811	0.984
	No (n=1)	2.7 (0, infinity)		
Hyperbilirubinemia	Yes (n=11)	10.1 (4.5-15.7)	0.86	0.039
	No (n=46)	12 (4.2-20)		
Venous thromboembolism	Yes (n=12)	12 (3.6-20.4)	0.593	0.281
	No (n=45)	13 (6.8-19.2)		
Body mass index (BMI) ≥30 kg/m^2^	Yes (n=11)	12 (0-26.9)	**0.05**	**0.023**
	No (n=46)	10.1 (4.2-16)		

**Figure 4:**
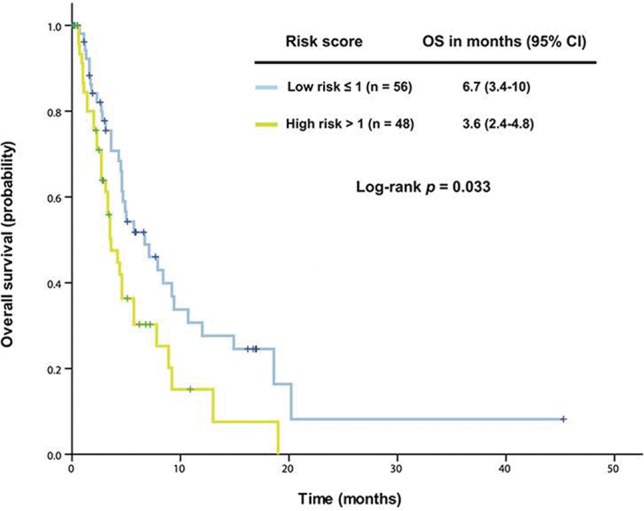
The established prognostic model was validated in 104 patients with advanced *KRAS+*/*TP53+* mutant cancer who did not receive therapy in a phase I clinical trial Kaplan-Meier overall survival (OS) curves are shown, stratified by risk score (low-risk group: score ≤1, high-risk group: score >1) (due to sample size, all p values are unadjusted).

To support this model, we used another cohort of patients who were referred to a phase I clinical trial but did not receive the therapy. In this cohort, patients in the low-risk group (n = 56) had a median OS of 6.7 months (95% CI 3.4-10.0), which was significantly better than that of those in the high-risk group (n = 48, median OS 3.6 months [95% CI 2.4-4.8], *p* = 0.033), as shown in Figure 4.

## DISCUSSION

Our findings suggest that the *KRAS* G13 and *TP53* R273 mutations are associated with poor outcome in patients with *KRAS*+/*TP53*+ mutant cancer, and antiangiogenic therapy combined with therapy targeting specific genetic aberrations may be an effective treatment strategy. To the best of our knowledge, the current study is the first to analyze clinical outcomes of patients with advanced hotspot *KRAS+*/*TP53+* mutant cancers who were referred to a phase I clinical trial program at MD Anderson.

*KRAS* and *TP53* are frequently mutated in many types of cancer. Although they are highly attractive therapeutic targets, they remain outside of the reach of direct pharmacologic intervention [20]. Until a breakthrough is achieved with a direct pharmacologic approach, alternative strategies for addressing these undruggable targets remain under investigation [24]. Unfortunately, we found that only approximately one-third of patients with advanced hotspot *KRAS+*/*TP53+* mutant cancers received treatment in a phase I clinical trial, much less than the overall rate of 55% of all patients who were referred to phase I clinical trials at the same institution [25].

A median OS of 12 months was observed in patients with *KRAS+*/*TP53+* mutant cancer who had received treatment in a phase I clinical trial, consistent with a previous study showing a median OS of 10 months in 1,181 consecutive cancer patients treated in phase I clinical trials [23]. Other studies have reported a median OS of 8 months in 365 patients harboring hotspot *KRAS* mutations [13] and 14.6 months in 188 patients harboring hotspot *TP53* mutations at the same institution [14]. These findings indicate that outcomes for patients with hotspot *KRAS+*/*TP53+* mutant cancer who enroll in phase I clinical trials are better than in those with hotspot *KRAS* mutations [13] but worse than in those with hotspot *TP53* mutations [14]. The differential outcomes for patients with specific cancer genetics [26] may reflect the reality that there are many phase I clinical trials of antiangiogenic-based therapeutic regimens but few studies appropriate for those with hotspot *KRAS* mutations [25]. These findings also suggest that patients harboring hotspot *TP53* mutations may benefit from antiangiogenic-based therapeutic regimens [19]. The evidence that the Royal Marsden Hospital score or the MD Anderson prognostic score could not be used to predict outcomes of the patients with KRAS+/TP53+ mutant cancer who received a phase I clinical trial therapy may indicate that the outcome was related to their unique biological characteristics, and availability of effective phase I trial therapy.

In our cohort of patients with *KRAS+*/*TP53+* mutant cancer, approximately two-thirds of patients had *KRAS* G12 mutations and one-sixth had G13 mutations. Although the absence of a G13 mutation is usually associated with poor prognosis in pancreatic cancer, the presence of a G13 mutation was associated with significantly shorter OS than other *KRAS* mutations in our full cohort of patients with *KRAS+*/*TP53+* mutations and in those with colorectal cancer. This is consistent with previous findings showing that the *KRAS* G13 mutation was an independent prognostic factor for poor metastasis-free survival in colon cancer compared with either wild-type *KRAS* or G12 mutation [27, 28].

We observed a total of 83 types of *TP53* mutations in our cohort, and most were located in the DNA binding domain. In contrast with a previous study showing that patients with hotspot *TP53* R273 mutant ovarian cancer had significantly longer median OS than those with other hotspot *TP53* mutations [29], our study revealed that a hotspot *TP53* R273 mutation was associated with poor survival in patients with metastatic colorectal cancer. These inconsistent data imply that different cell contexts may lead to different outcomes, which warrants further investigation clinically and preclinically.

Although genetics likely play an important role in tumorigenesis, the inflammatory process is initiated by the movement of innate immune system cells to the microenvironment, followed by the secretion of proinflammatory cytokines, growth factors, and reactive oxygen species, causing DNA damage and promoting neoplastic development, as has been found in many tumor types [6]. Our multivariable analysis revealed that five independent baseline factors (neutrophilia, thrombocytosis, hypoalbuminemia, body mass index <30 kg/m^2^, and the absence of lung metastasis) were able to predict individual outcome not only in patients with *KRAS+*/*TP53+* mutant cancer who had received therapy in a phase I clinical trial, but also in those who had not received therapy. Four of these prognostic factors are related to the proinflammatory state, which works alongside *KRAS* and *TP53* mutations to enhance tumor progression and develop resistance to cancer therapy, resulting in poor clinical outcomes [30–32]. Therefore, a thorough understanding of the mechanisms of the proinflammatory state in conjunction with cancer-related gene aberrations may provide a scientific rationale to develop effective therapeutic strategies for advanced *KRAS+*/*TP53+* mutant cancer. Though we cannot completely explain association of the absence of lung metastasis with poor outcome, we did observe that phase I metastatic colorectal cancer patients with pulmonary metastasis had a relatively slow process for tumor progression, which might reflect different biologic properties of these tumors, and requires further investigation.

Our study has limitations. First, the retrospective setup and limited sample size might yield statistical bias. Due to multiplicity of statistical testing in such small sample size, all *p* values are exploratory and unadjusted. Second, data from patients with hotspot *KRAS+*/*TP53*-, *KRAS*-/*TP53+*, and *KRAS*-/*TP53*- cancer were not available for our analysis of patients with metastatic *KRAS+*/*TP53+* mutant cancer, which limited our ability to reach conclusions from data comparison among these four groups of patients.

## PATIENTS AND METHODS

### Patients

We retrospectively reviewed 2,144 consecutive patients with advanced cancers who were referred to phase I clinical trials at MD Anderson from March 2102 to October 2014 and who had sufficient tumor tissue specimens available for next generation sequencing. Among these patients, 167 harbored concurrent hotspot mutations in the *KRAS* and *TP53* genes. Patient baseline demographics, laboratory results, gene aberrations, status of phase I clinical trial therapy, and clinical outcomes were obtained from electronic medical records. All patients were followed until death or censored on March 10, 2016. Trial conduct, data collection, and subsequent data analysis were performed in accordance with the guidelines of the MD Anderson Institutional Review Board (IRB) after the IRB approval for the research and a waiver of informed consent were obtained.

### Molecular analysis

For somatic hotspot mutation analysis, DNA was extracted, purified, and quantified from microdissected, paraffin-embedded tumor specimens. Next generation sequencing for hotspot mutations was performed using the Ion Ampliseq Cancer Panel (Life Technologies, Grand Island, NY) in a Clinical Laboratory Improvement Amendments-certified Molecular Diagnostics Laboratory at MD Anderson [19, 33]. A panel of 46 genes was initially tested and then expanded to 50 genes, as described previously [34].

### Treatment and evaluation

The decision to enroll an eligible patient in a phase I clinical trial depended on the protocol availability and the discretion of the treating physicians. Tumor responses (CR = complete remission, PR = partial response, SD = stable disease, and PD = progressive disease) were evaluated according to Response Evaluation Criteria in Solid Tumors (RECIST) version 1.0 or 1.1 [35, 36], depending on individual protocols. Progression-free survival (PFS) was calculated from the date of initiation of a phase I clinical trial therapy to the date of first objective documentation of PD, death, or censor date. PFS for patients alive and progression-free at last evaluation should be censored at date of last clinical evaluation. Overall survival (OS) was calculated from the date of the initial phase I clinical trial visit to the date of death or censor date. Time to death for patients alive at last contact should be censored at date of last contact.

### Statistical analysis

Continuous interval-scaled data were summarized as median (range). Categorical data were summarized as frequencies and relative frequencies. Associations between categorical variables were tested using the chi-squared and Fisher exact tests. PFS and OS curves were estimated using the Kaplan-Meier method and compared using log rank tests. Cox proportional hazards regression analysis was used for multivariable analysis. All tests were two-sided and considered significant when p < 0.05. Analyses were performed using SPSS version 23.0 (SPSS, Chicago, IL).

## CONCLUSIONS

We found that hotspot *KRAS+*/*TP53+* mutations occurred in approximately 8% of cancer patients referred to our institution for phase I clinical trials, and that the *KRAS* G13 mutation, as well as the *TP53* R273 mutation, were associated with poor OS. Antiangiogenesis and gene aberration-related therapies may improve overall survival in patients with concurrent *KRAS+*/*TP53+* hotspot mutant cancer. Also our data also suggest that the proinflammatory state is a key event in cancer development, facilitated through evolving gene aberrations. The current study has provided further support that the combination of modulating the proinflammatory state via immunotherapeutic agents [37] with expanding pharmacologic manipulation to address undruggable molecular cancer targets may lead to novel and effective approaches to the treatment of *KRAS+*/*TP53+* mutant advanced cancer.

## References

[1] Cox AD, Fesik SW, Kimmelman AC, Luo J, Der CJ (2014). Drugging the undruggable RAS: Mission possible?. Nature reviews Drug discovery.

[2] Kandoth C, McLellan MD, Vandin F, Ye K, Niu B, Lu C, Xie M, Zhang Q, McMichael JF, Wyczalkowski MA, Leiserson MD, Miller CA, Welch JS (2013). Mutational landscape and significance across 12 major cancer types. Nature.

[3] Singh H, Longo DL, Chabner BA (2015). Improving Prospects for Targeting RAS. Journal of clinical oncology.

[4] Okumura S, Janne PA (2014). Molecular pathways: the basis for rational combination using MEK inhibitors in KRAS-mutant cancers. Clinical cancer research.

[5] Tsuchida N, Murugan AK, Grieco M (2016). Kirsten Ras* oncogene: Significance of its discovery in human cancer research. Oncotarget.

[6] Szylberg L, Janiczek M, Popiel A, Marszalek A (2015). Large Bowel Genetic Background and Inflammatory Processes in Carcinogenesis—Systematic Review. Advances in clinical and experimental medicine.

[7] O’Dell MR, Huang JL, Whitney-Miller CL, Deshpande V, Rothberg P, Grose V, Rossi RM, Zhu AX, Land H, Bardeesy N, Hezel AF (2012). Kras(G12D) and p53 mutation cause primary intrahepatic cholangiocarcinoma. Cancer research.

[8] Chow OS, Kuk D, Keskin M, Smith JJ, Camacho N, Pelossof R, Chen CT, Chen Z, Avila K, Weiser MR, Berger MF, Patil S, Bergsland E (2016). KRAS and combined KRAS/TP53 mutations in locally advanced rectal cancer are independently associated with decreased response to neoadjuvant therapy. Annals of surgical oncology.

[9] Rivlin N, Brosh R, Oren M, Rotter V (2011). Mutations in the p53 tumor suppressor gene: important milestones at the various steps of tumorigenesis. Genes Cancer.

[10] Bigi A, Beltrami E, Trinei M, Stendardo M, Pelicci PG, Giorgio M (2016). Cyclophilin D counteracts P53-mediated growth arrest and promotes Ras tumorigenesis. Oncogene.

[11] Cancer Genome Atlas Research N (2014). Comprehensive molecular profiling of lung adenocarcinoma. Nature.

[12] Bailey P, Chang DK, Nones K, Johns AL, Patch AM, Gingras MC, Miller DK, Christ AN, Bruxner TJ, Quinn MC, Nourse C, Murtaugh LC, Harliwong I (2016). Genomic analyses identify molecular subtypes of pancreatic cancer. Nature.

[13] Said R, Ye Y, Falchook GS, Janku F, Naing A, Zinner R, Blumenschein GR, Fu S, Hong DS, Piha-Paul SA, Wheler JJ, Kurzrock R, Palmer GA (2014). Outcomes of patients with advanced cancer and KRAS mutations in phase I clinical trials. Oncotarget.

[14] Said R, Ye Y, Hong DS, Janku F, Fu S, Naing A, Wheler JJ, Kurzrock R, Thomas C, Palmer GA, Hess KR, Aldape K, Tsimberidou AM (2014). Characteristics and survival of patients with advanced cancer and p53 mutations. Oncotarget.

[15] Acin S, Li Z, Mejia O, Roop DR, El-Naggar AK, Caulin C (2011). Gain-of-function mutant p53 but not p53 deletion promotes head and neck cancer progression in response to oncogenic K-ras. The Journal of pathology.

[16] Ji H, Ramsey MR, Hayes DN, Fan C, McNamara K, Kozlowski P, Torrice C, Wu MC, Shimamura T, Perera SA, Liang MC, Cai D, Naumov GN (2007). LKB1 modulates lung cancer differentiation and metastasis. Nature.

[17] Hunter SM, Anglesio MS, Ryland GL, Sharma R, Chiew YE, Rowley SM, Doyle MA, Li J, Gilks CB, Moss P, Allan PE, Stephens AN, Huntsman DG (2015). Molecular profiling of low grade serous ovarian tumours identifies novel candidate driver genes. Oncotarget.

[18] Hou MM, Wang Z, Janku F, Piha-Paul S, Naing A, Hong D, Westin S, Coleman RL, Sood AK, Tsimberidou AM, Subbiah V, Wheler J, Zinner R (2016). Continuous anti-angiogenic therapy after tumor progression in patients with recurrent high-grade epithelial ovarian cancer: phase I trial experience. Oncotarget.

[19] Fu S, Hou MM, Naing A, Janku F, Hess K, Zinner R, Subbiah V, Hong D, Wheler J, Piha-Paul S, Tsimberidou A, Karp D, Araujo D (2015). Phase I study of pazopanib and vorinostat: a therapeutic approach for inhibiting mutant p53-mediated angiogenesis and facilitating mutant p53 degradation. Annals of oncology.

[20] Lazo JS, Sharlow ER (2016). Drugging Undruggable Molecular Cancer Targets. Annual review of pharmacology and toxicology.

[21] Hantschel O, Grebien F, Superti-Furga G (2011). Targeting allosteric regulatory modules in oncoproteins: “drugging the undruggable”. Oncotarget.

[22] Arkenau HT, Barriuso J, Olmos D, Ang JE, de Bono J, Judson I, Kaye S (2009). Prospective validation of a prognostic score to improve patient selection for oncology phase I trials. Journal of clinical oncology.

[23] Wheler J, Tsimberidou AM, Hong D, Naing A, Falchook G, Piha-Paul S, Fu S, Moulder S, Stephen B, Wen S, Kurzrock R (2012). Survival of 1,181 patients in a phase I clinic: the MD Anderson Clinical Center for targeted therapy experience. Clinical cancer research.

[24] Chen Z, Cheng K, Walton Z, Wang Y, Ebi H, Shimamura T, Liu Y, Tupper T, Ouyang J, Li J, Gao P, Woo MS, Xu C (2012). A murine lung cancer co-clinical trial identifies genetic modifiers of therapeutic response. Nature.

[25] Fu S, McQuinn L, Naing A, Wheler JJ, Janku F, Falchook GS, Piha-Paul SA, Tu D, Howard A, Tsimberidou A, Zinner R, Hong DS, Kurzrock R (2013). Barriers to study enrollment in patients with advanced cancer referred to a phase I clinical trials unit. The oncologist.

[26] Janku F, Hong DS, Fu S, Piha-Paul SA, Naing A, Falchook GS, Tsimberidou AM, Stepanek VM, Moulder SL, Lee JJ, Luthra R, Zinner RG, Broaddus RR (2014). Assessing PIK3CA and PTEN in early-phase trials with PI3K/AKT/mTOR inhibitors. Cell reports.

[27] Peeters M, Douillard JY, Van Cutsem E, Siena S, Zhang K, Williams R, Wiezorek J (2013). Mutant KRAS codon 12 and 13 alleles in patients with metastatic colorectal cancer: assessment as prognostic and predictive biomarkers of response to panitumumab. Journal of clinical oncology.

[28] Ilm K, Kemmner W, Osterland M, Burock S, Koch G, Herrmann P, Schlag PM, Stein U (2015). High MACC1 expression in combination with mutated KRAS G13 indicates poor survival of colorectal cancer patients. Molecular cancer.

[29] Seagle BL, Yang CP, Eng KH, Dandapani M, Odunsi-Akanji O, Goldberg GL, Odunsi K, Horwitz SB, Shahabi S (2015). TP53 hot spot mutations in ovarian cancer: selective resistance to microtubule stabilizers in vitro and differential survival outcomes from The Cancer Genome Atlas. Gynecologic oncology.

[30] Serresi M, Gargiulo G, Proost N, Siteur B, Cesaroni M, Koppens M, Xie H, Sutherland KD, Hulsman D, Citterio E, Orkin S, Berns A, van Lohuizen M (2016). Polycomb Repressive Complex 2 Is a Barrier to KRAS-Driven Inflammation and Epithelial-Mesenchymal Transition in Non-Small-Cell Lung Cancer. Cancer cell.

[31] Aguilera-Aguirre L, Bacsi A, Radak Z, Hazra TK, Mitra S, Sur S, Brasier AR, Ba X, Boldogh I (2014). Innate inflammation induced by the 8-oxoguanine DNA glycosylase-1-KRAS-NF-kappaB pathway. Journal of immunology.

[32] Pal S, Bhattacharjee A, Ali A, Mandal NC, Mandal SC, Pal M (2014). Chronic inflammation and cancer: potential chemoprevention through nuclear factor kappa B and p53 mutual antagonism. Journal of inflammation.

[33] Hou MM, Liu X, Wheler J, Naing A, Hong D, Coleman RL, Tsimberidou A, Janku F, Zinner R, Lu K, Kurzrock R, Fu S (2014). Targeted PI3K/AKT/mTOR therapy for metastatic carcinomas of the cervix: A phase I clinical experience. Oncotarget.

[34] Meric-Bernstam F, Brusco L, Shaw K, Horombe C, Kopetz S, Davies MA, Routbort M, Piha-Paul SA, Janku F, Ueno N, Hong D, De Groot J, Ravi V (2015). Feasibility of large-scale genomic testing to facilitate enrollment onto genomically matched clinical trials. Journal of clinical oncology.

[35] Therasse P, Arbuck SG, Eisenhauer EA, Wanders J, Kaplan RS, Rubinstein L, Verweij J, Van Glabbeke M, van Oosterom AT, Christian MC, Gwyther SG (2000). New guidelines to evaluate the response to treatment in solid tumors. European Organization for Research and Treatment of Cancer, National Cancer Institute of the United States, National Cancer Institute of Canada. J Natl Cancer Inst.

[36] Eisenhauer EA, Therasse P, Bogaerts J, Schwartz LH, Sargent D, Ford R, Dancey J, Arbuck S, Gwyther S, Mooney M, Rubinstein L, Shankar L, Dodd L (2009). New response evaluation criteria in solid tumours: revised RECIST guideline (version 1.1). Eur J Cancer.

[37] Baniyash M, Sade-Feldman M, Kanterman J (2014). Chronic inflammation and cancer: suppressing the suppressors. Cancer immunology, immunotherapy.

